# Targeting the OTU family: a core therapeutic strategy for reshaping the immunosuppressive microenvironment and reversing drug resistance in HCC by coordinating the autophagy-ferroptosis balance

**DOI:** 10.1038/s41420-026-03148-1

**Published:** 2026-05-05

**Authors:** Pengcheng Zhao, Ping Zhang

**Affiliations:** 1 Department of Hepatobiliary Pancreas and Spleen Surgery, General Surgery Center, the First Hospital of Jilin University (the First Bethune Hospital of Jilin University), Changchun, China; 2 Jilin University, Changchun, China

**Keywords:** Hepatocellular carcinoma, Hepatocellular carcinoma, Tumour immunology

## Abstract

Hepatocellular carcinoma (HCC) treatment faces dual challenges: resistance to targeted therapy and low response rates to immunotherapy. These issues are rooted in the immunosuppressive tumor microenvironment (TME). Ferroptosis and autophagy, two critical cellular processes, play complex and paradoxical roles in HCC drug resistance and immunoregulation, and they interact closely. This review explores how the OTU deubiquitinase family, especially OTUB1, acts as a central hub coordinating the autophagy-ferroptosis balance. Additionally, other OTU family members, such as OTUD3, OTULIN, and OTUD6B, contribute to HCC progression by modulating similar pathways, highlighting the need for a broader therapeutic approach. Specifically, OTUD3 suppresses HIF-1α-driven angiogenesis, OTULIN inhibits NF-κB-mediated inflammation, and OTUD6B stabilizes pVHL to impede metastasis, collectively demonstrating their synergistic or antagonistic interactions with OTUB1 in reshaping the TME. This coordination drives HCC drug resistance and remodels the immune microenvironment. OTUB1 suppresses ferroptosis and maintains tumor cell survival by deubiquitinating and stabilizing key proteins like SLC7A11, GPX4, and p62. It also promotes immune escape by modulating PD-L1 stability and immune cell function. Consequently, therapeutic strategies targeting the OTU family—such as developing selective inhibitors for multiple members, using intelligent nanodelivery systems, and combining them with ferroptosis inducers or immune checkpoint inhibitors—show significant potential for reversing drug resistance and improving immunotherapy efficacy. Expanding these strategies to include other OTU members could enhance efficacy and reduce resistance. Addressing how the OTU family precisely modulates the intersection of autophagy and ferroptosis, and how it reshapes immune cell metabolism and function within the TME, is critical for developing novel combination therapies. This article provides a crucial theoretical foundation for developing novel combination strategies targeting metabolism-immune crosstalk.

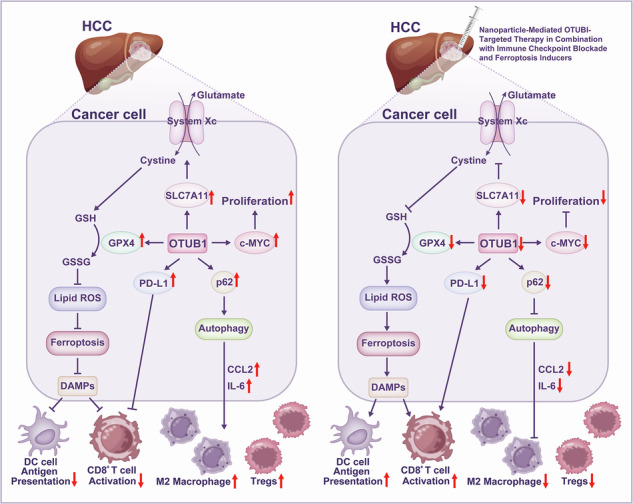

## Facts

- OTUB1 is upregulated across diverse solid tumors and correlates with poor prognosis.
- By deubiquitinating oncogenic proteins such as HIF-1α, RACK1, and PD-L1, OTUB1 drives tumor growth and immune evasion.
- OTUB1 suppresses ferroptosis by stabilizing GPX4 and SLC7A11, fostering chemoresistance.
- Ferritinophagy releases labile iron and amplifies K48-, Ferroptosis signaling.
- Targeting OTUB1 restores drug sensitivity and represents a promising therapeutic avenue in hepatocellular carcinoma.

## Open questions

- How exactly does OTUB1 modulate autophagy in hepatocellular carcinoma, and where does this intersect with ferroptosis regulation?
- In what ways does OTUB1 reshape immune cell function and metabolism within the tumor microenvironment?
- Can OTUB1 blockade synergize with ferroptosis inducers or immune checkpoint inhibitors for greater therapeutic efficacy?
- How can highly selective OTUB1 inhibitors and predictive biomarkers be developed to enable precision treatment in hepatocellular carcinoma?

## Introduction: The intertwined challenges of drug resistance and the immunosuppressive microenvironment in HCC

Hepatocellular carcinoma (HCC) remains a leading cause of cancer-related mortality worldwide, with limited therapeutic advancements despite the introduction of molecular targeted agents and immune checkpoint inhibitors (ICIs). The persistent challenges of intrinsic and acquired drug resistance, coupled with a low response rate to immunotherapy (approximately 15–20% with ICI monotherapy), underscore the urgent need to understand the underlying mechanisms driving therapeutic failure [1–5]. Increasingly, evidence points to the immunosuppressive tumor microenvironment (TME) as a critical nexus where metabolic reprogramming, altered cell death pathways, and immune evasion converge to sustain HCC progression [6–9].

A key open question is how specific regulators, such as the OTU deubiquitinase OTUB1, integrate these processes to foster therapy resistance. Within the TME, immunosuppressive cells—including tumor-associated macrophages (TAMs), regulatory T cells (Tregs), and myeloid-derived suppressor cells (MDSCs)—secrete factors that blunt effective immune responses and promote resistance to treatments such as anti-PD-1 therapy [10]. Simultaneously, tumor cells adapt to therapeutic pressure through metabolic alterations, including dysregulated iron homeostasis and lipid metabolism, further reinforcing a treatment-resistant niche [2, 5]. Central to this adaptive response are two interconnected cellular processes: ferroptosis and autophagy. Ferroptosis, an iron-dependent form of regulated cell death driven by lipid peroxidation, exhibits dual immunomodulatory roles. It can promote immunogenic cell death (ICD) and enhance antitumor immunity by releasing DAMPs such as HMGB1 and ATP [11, 12]. Conversely, it may also suppress immune function under certain conditions [13, 14]. Autophagy similarly plays a context-dependent role, supporting tumor cell survival under stress while potentially impairing antigen presentation and recruiting immunosuppressive stromal cells when dysregulated [15–18]. The delicate balance between ferroptosis and autophagy, and their collective impact on immune activation or suppression, represents a fundamental axis influencing HCC treatment outcomes. The precise molecular mechanisms by which this balance is coordinated, particularly by deubiquitinating enzymes (DUBs), and how it can be therapeutically targeted, remain active areas of investigation.

The OTU deubiquitinase family, particularly OTUB1, has emerged as a master regulator at the intersection of autophagy, ferroptosis, and immune modulation. By stabilizing key proteins such as SLC7A11, GPX4, and p62, OTUB1 directly suppresses ferroptosis and fine-tunes autophagic activity, thereby enabling tumor cells to evade lethal stress [19, 20]. In parallel, OTU members regulate immune cell functions—including T cell activation, macrophage polarization, and dendritic cell maturation—through deubiquitination of signaling intermediates such as TRAF6, PD-L1, and CARD9 [21–23]. This positions the OTU family as a central coordinator of the autophagy-ferroptosis balance and a determinant of the immune landscape in HCC. Understanding the full scope of OTUB1’s impact on immune cell metabolism and function within the HCC TME is essential for designing effective combination immunotherapies.

This review explores how targeting the OTU family, especially OTUB1, offers a promising strategy to disrupt the self-reinforcing cycle of drug resistance and immunosuppression. Graphical Abstract summarizes the therapeutic mechanisms, illustrating OTUB1’s role in HCC progression and treatment. By systematically dissecting the roles of OTU-mediated ubiquitination signaling in coordinating metabolic stress responses and immune evasion, we aim to provide a translational framework for novel combination therapies that simultaneously sensitize HCC to ferroptosis and enhance immunotherapy efficacy. A central theme will be to evaluate the potential synergy between OTUB1 blockade and existing modalities like ferroptosis inducers or ICIs, and to discuss the challenges in developing highly selective OTUB1 inhibitors and predictive biomarkers for precision medicine.

## Functional characteristics of the OTU deubiquitinase family and their relevance in cancer

### Functional classification and dual roles in tumor regulation of the OTU family

The OTU (Ovarian Tumor) deubiquitinase family belongs to the cysteine protease class of DUBs. Its defining feature is the presence of a highly conserved OTU domain, which facilitates the hydrolysis of ubiquitin chains via a catalytic triad (Cys/His/Asp), thereby reversing protein ubiquitination [21]. Based on differences in substrate specificity and structure, the OTU family can be classified into four subfamilies: the OTUB subfamily (e.g., OTUB1, OTUB2), the OTUD subfamily (e.g., OTUD1, OTUD3, OTUD5/YOD1, OTUD6A/B), the A20-like subfamily (e.g., A20/TNFAIP3, Cezanne), and the OTULIN subfamily [23]. Individual members participate in key biological processes—such as proteasomal degradation, NF-κB signal transduction, inflammatory responses, and DNA damage repair—through selective cleavage of specific ubiquitin chain types (e.g., K48-, K63-, or linear-linked chains) [24].

In cancer regulation, the OTU family exhibits marked functional duality: on one hand, certain members such as OTUD1 (YOD1) and OTUD6A promote tumor proliferation, metastasis, and chemotherapy resistance by deubiquitinating and stabilizing oncoproteins (e.g., CDK1, TopBP1) [25]; on the other hand, others such as OTUD3 and OTULIN act as tumor suppressors by inhibiting pro-oncogenic signaling pathways (e.g., HIF-1α or NF-κB) [26]. For instance, in HCC, OTUD3 has been shown to suppress tumor growth by destabilizing HIF-1α, thereby reducing angiogenesis, while OTULIN inhibits NF-κB signaling to mitigate inflammation-driven resistance [26, 27]. OTUD6B, another member, suppresses HIF-2α-mediated angiogenesis and metastasis by deubiquitinating and stabilizing the pVHL protein [27], whereas OTUB1 enhances the stability of the JAK2/STAT1 signaling pathway to promote invasion and migration in glioblastoma (GBM) cells [28]. This functional diversity stems from differences in substrate specificity and variations in expression profiles across different cancer types, underscoring the importance of considering the entire OTU family in HCC therapeutic strategies.

### OTU family in immune regulation: a systematic overview of functions across immune cell subsets

The OTU deubiquitinase family plays pivotal roles in regulating immune cell development, activation, and functional polarization, thereby shaping antitumor immunity. Different OTU members exhibit cell-type-specific functions, forming a complex regulatory network within the immune microenvironment. Beyond OTUB1, members like OTUD5 and OTUD6B directly influence immune responses in HCC; for example, OTUD5 promotes M2 macrophage polarization via CARD9 stabilization, contributing to immunosuppression, while OTUD6B enhances CD8^+^ T cell recruitment in colorectal cancer models, suggesting potential cross-application in HCC [29, 30]. Importantly, OTUD3 and OTULIN also modulate immune cell activity: OTUD3 destabilizes HIF-1α to reduce angiogenesis-associated immune suppression [26], and OTULIN restrains NLRP3 inflammasome activation to limit pro-inflammatory polarization [31]. These findings underscore that the OTU family functions as an interconnected system, where targeting multiple members may disrupt immune evasion more effectively than focusing solely on OTUB1.

#### Regulation of T cell activation and exhaustion

**Fig. 1:**
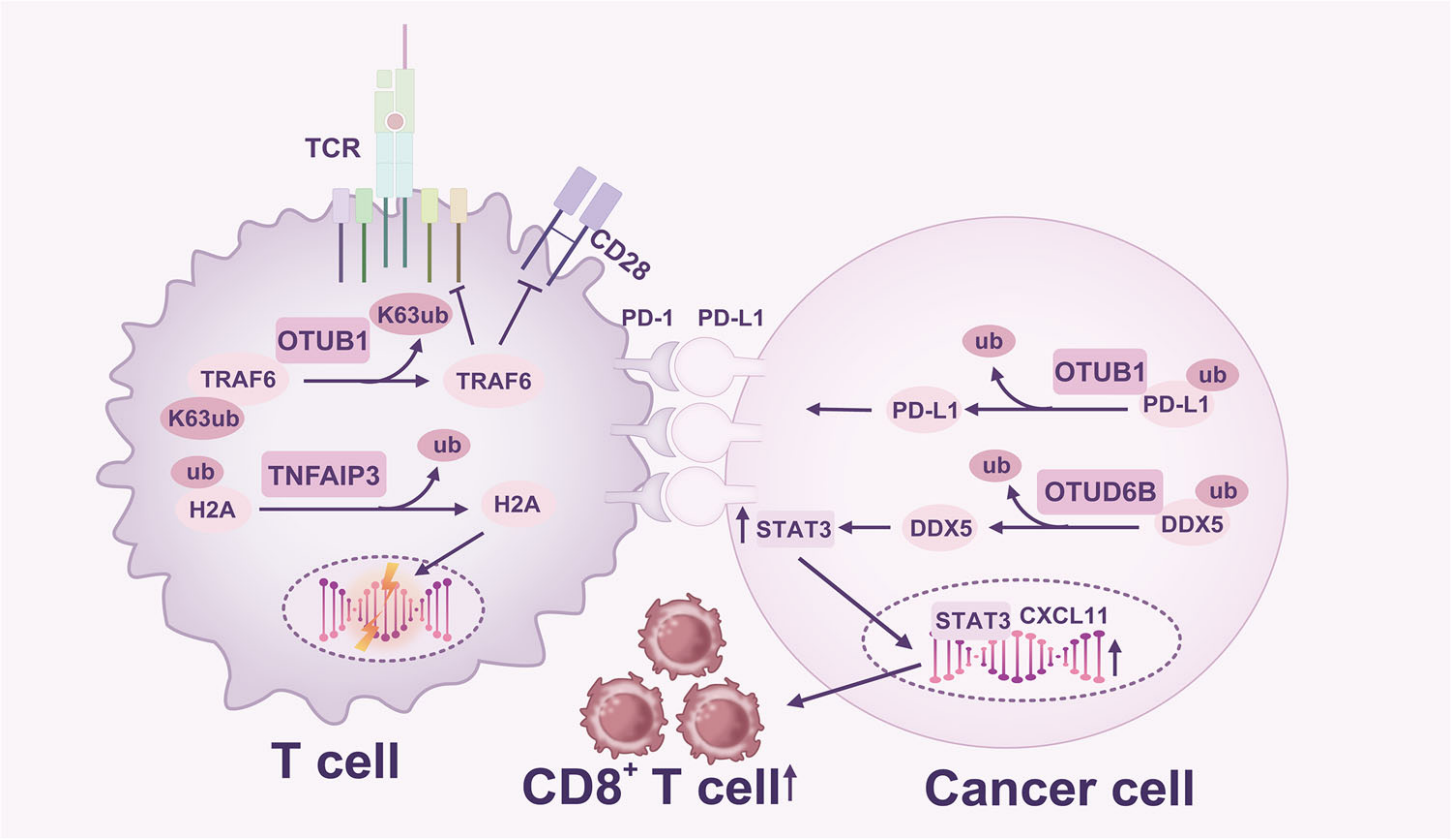
Functions of the OTU family in T cells. This schematic illustrates the molecular mechanisms within the tumor immune microenvironment. In CD8⁺ T cells (left), OTUB1 deubiquitinates and stabilizes TRAF6, enhancing K63-linked ubiquitination (K63-ub) signaling pathways that promote T cell activation. Conversely, in cancer cells (right), OTUB1 deubiquitinates and stabilizes PD-L1, increasing its surface expression. The interaction between PD-1 (on T cells) and PD-L1 (on cancer cells) transmits an inhibitory signal that suppresses T cell function. Inhibition of OTUB1 (center) reduces the stability of both TRAF6 and PD-L1, thereby potentiating CD8⁺ T cell activation and cytotoxic function against cancer cells.

The OTU family profoundly influences T cell activation and exhaustion by modulating the ubiquitination status of key nodes in the T cell receptor (TCR) signaling pathway. For example, OTUB1 cleaves K63-linked ubiquitin chains on TRAF6, thereby inhibiting TCR–CD28 costimulatory signaling and limiting excessive T cell activation [32, 33]. It has been reported in non-small cell lung cancer that OTUB1 can influence the PD-1/PD-L1 signaling axis through deubiquitination of PD-L1, suggesting its involvement in shaping the T cell exhaustion phenotype [34]. On the other hand, OTUD6B recruits and activates CD8⁺ T cells via the DDX5–STAT3–CXCL11 signaling axis, thereby suppressing colorectal cancer liver metastasis [29]. Additionally, the deubiquitinase A20 (TNFAIP3) has been shown to indirectly regulate T cell function by modulating histone H2A ubiquitination and affecting DNA damage response [35] (see Fig. 1).

#### Control of macrophage polarization

**Fig. 2:**
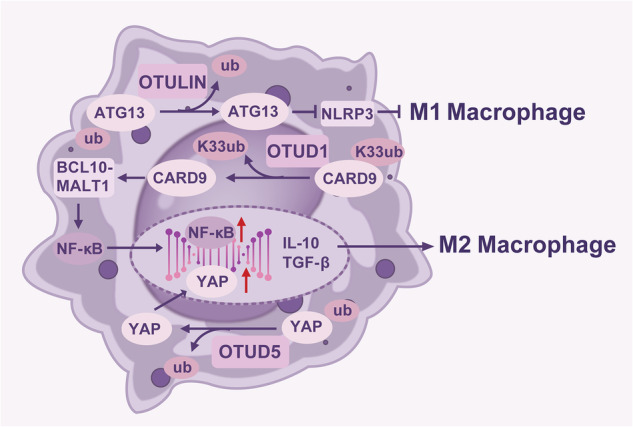
Functions of the OTU family in macrophage cells. This schematic illustrates the molecular mechanisms by which deubiquitinating enzymes (OTULIN, OTUD1, OTUD5) specifically regulate the ubiquitination of distinct proteins, thereby influencing macrophage polarization towards the pro-inflammatory M1 phenotype or the anti-inflammatory/reparative M2 phenotype. On the left, within the M1-type macrophage, OTULIN inhibits inflammasome activation by deubiquitinating NLRP3, while OTUD1 negatively regulates the CARD9-BCL10-MALT1 signalosome by cleaving K33-linked polyubiquitin chains (K33ub), consequently suppressing the NF-κB pathway; together, these actions restrict M1 polarization and the pro-inflammatory response. On the right, within the M2-type macrophage, OTUD5 stabilizes CARD9 via deubiquitination, thereby activating the NF-κB pathway and promoting the expression of anti-inflammatory factors such as IL-10 and TGF-β; simultaneously, the YAP signaling pathway is activated to foster M2 polarization. Furthermore, OTUD1 influences the autophagy process by regulating the deubiquitination of ATG13, and OTUD5 is also involved in modulating the YAP signaling pathway. This diagram reveals the core role of the deubiquitinating enzyme network as precise “molecular switches” that control the stability of key signaling molecules, central to orchestrating immune responses in macrophages.

Members of the OTU family influence the polarization direction of macrophages by modulating the stability of key signaling proteins. OTUD1 directly binds to the CARD9 protein and removes its K33-linked ubiquitin chains. This not only stabilizes CARD9 but also promotes the assembly of the CARD9–BCL10–MALT1 (CBM) signalosome complex, thereby strongly activating the NF-κB signaling pathway and triggering robust production of inflammatory cytokines [36]. In contrast, OTULIN suppresses NLRP3 inflammasome activation by hydrolyzing linear ubiquitin chains on ATG13, consequently restraining M1 pro-inflammatory polarization [31]. Furthermore, OTUD5 deubiquitinates and stabilizes the transcriptional coactivator YAP, preventing its proteasomal degradation. The accumulated YAP translocates into the nucleus and initiates the expression of genes associated with M2 polarization, such as IL-10, TGF-β, and VEGFA. This regulatory mechanism is particularly critical in the TME, where M2-polarized macrophages promote immune escape and tumor progression through the secretion of factors including IL-10 and TGF-β [30] (see Fig. 2).

#### Modulation of dendritic cell immune responses

**Fig. 3:**
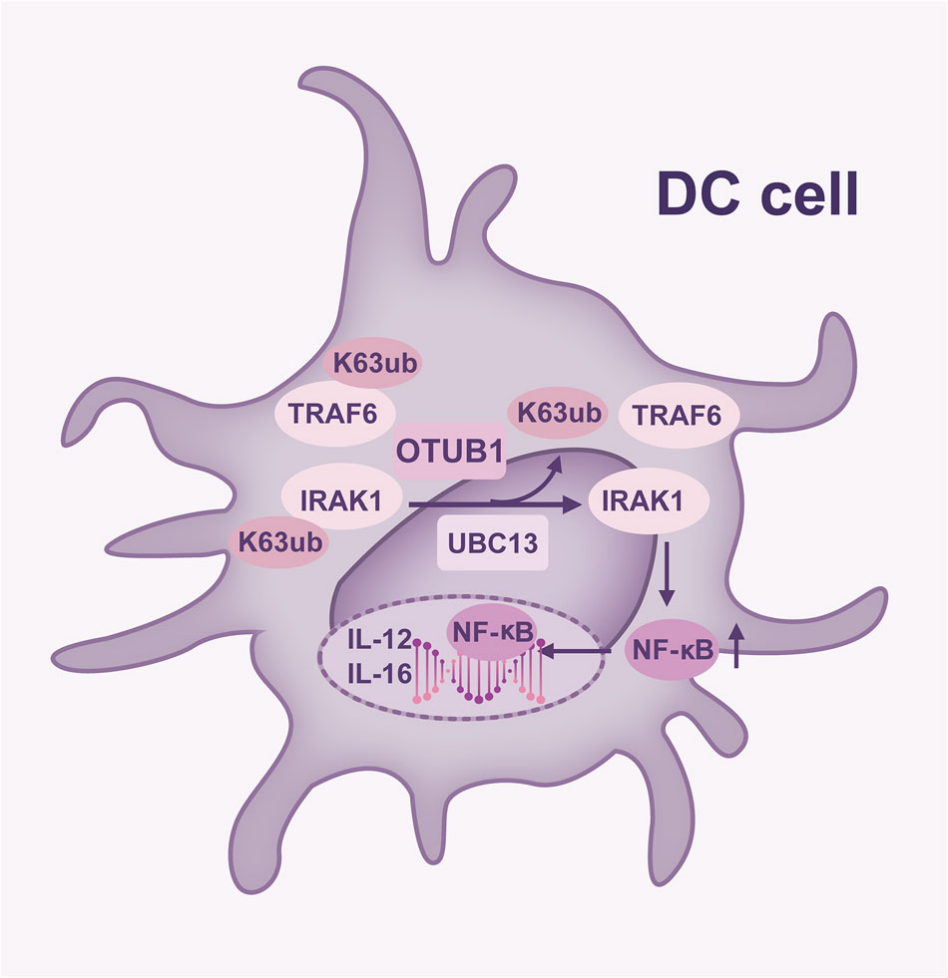
Functions of the OTU family in dendritic cells. This schematic illustrates the negative regulatory role of the deubiquitinating enzyme OTUB1 in the TLR/IL-1R signaling pathway within dendritic cells (DCs). In the canonical activation pathway (indicated by dashed arrows), upstream signals induce the E2 ubiquitin-conjugating enzyme UBC13 to form a complex with the adaptor protein TRAF6, catalyzing the K63-linked polyubiquitination (K63ub) of TRAF6. This modification serves as a molecular platform to recruit and activate the kinase IRAK1, which subsequently triggers the activation and nuclear translocation of the NF-κB transcription factor, ultimately driving the gene expression of pro-inflammatory cytokines such as IL-12 and IL-16. OTUB1 (shown in purple) negatively regulates this signaling cascade through its interactions with both IRAK1 and UBC13. OTUB1 likely attenuates IRAK1 activation by removing the K63ub modification and/or disrupting the complex formation, thereby dampening the NF-κB signaling pathway and limiting the immunostimulatory functions of DCs.

The activation, cytokine production, and immune functions of dendritic cells (DCs) are finely regulated by the OTU family. OTUB1 promotes NF-κB activity in DCs through K48-linked deubiquitination and stabilization of the E2 conjugating enzyme UBC13, leading to enhanced K63-linked ubiquitination of IRAK1 and TRAF6. This subsequently drives the production of pro-inflammatory cytokines such as IL-12, IL-6, and TNF [37]. Beyond OTUB1, other OTU members also contribute to DC regulation: OTUD7B modulates mTORC2 complex assembly by deubiquitinating GβL (MLST8), thereby indirectly influencing DC metabolism and function [38]; meanwhile, OTUD6B regulates protein translation processes through its different isoforms (OTUD6B-1 and OTUD6B-2), which indirectly affect DC proliferation and functionality [39] (see Fig. 3).

## Regulatory roles of OTUB1 in the immune microenvironment

As a key member of the deubiquitinase family, OTUB1 plays a central role in shaping the tumor immune microenvironment (TIME) through its dual regulatory functions in both tumor cells and immune cells. Its regulatory network is extensive and complex, profoundly influencing the efficacy of antitumor immune responses. A significant gap in knowledge concerns the precise ways OTUB1 reshapes immune cell function and metabolism within the HCC TME.

### Indirect regulation of immune responses by tumor cell–intrinsic OTUB1

**Fig. 4:**
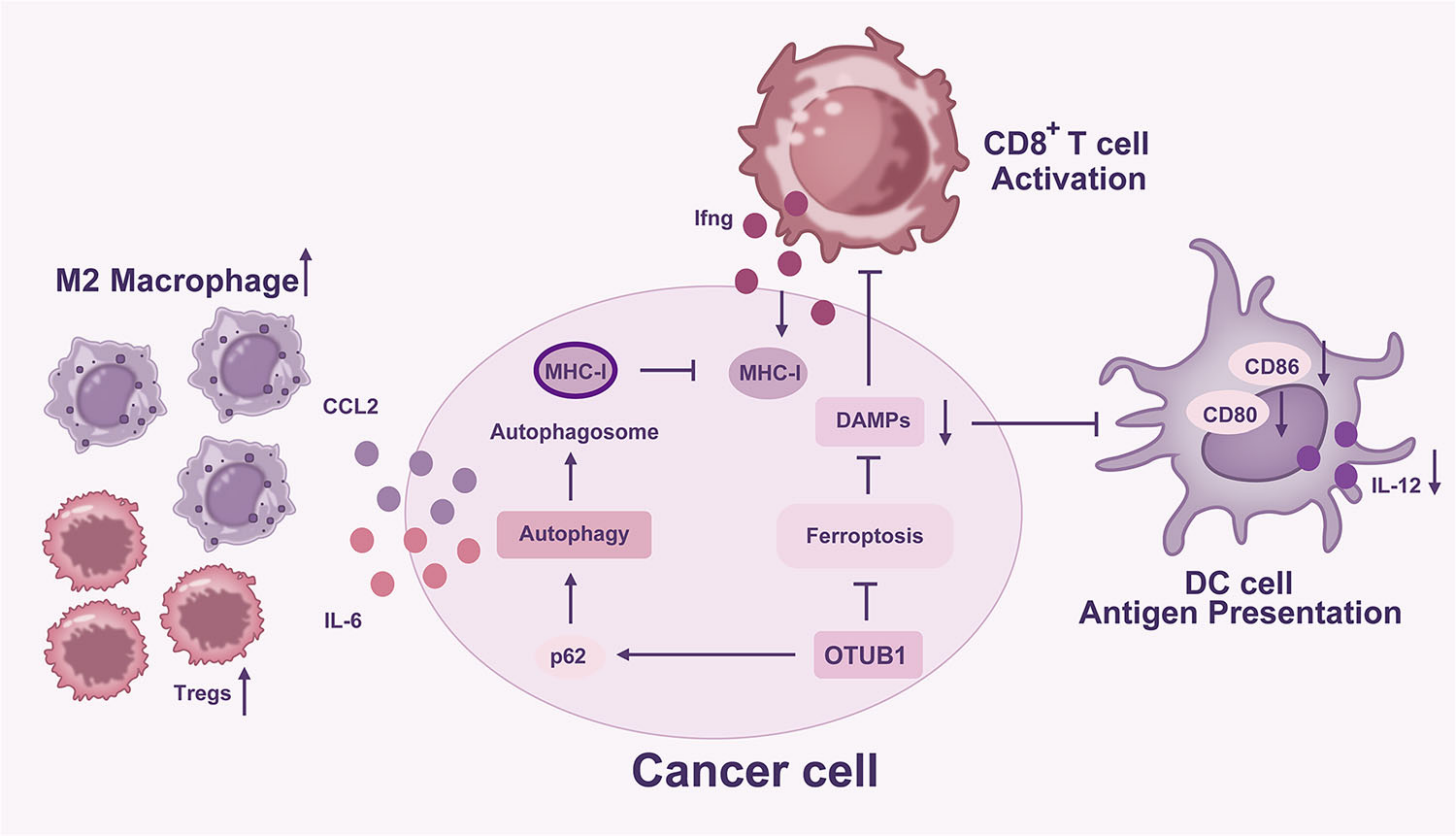
Indirect regulation of immune responses by tumor cell–intrinsic OTUB1. This schematic provides a holistic overview of the complex interaction network between cancer cells and different types of immune cells within the tumor microenvironment (TME), highlighting the dynamic balance between immune activation and immune escape. The core processes include: (1) Immune Activation Pathway: Dendritic cells (DCs) present antigen via MHC-I and provide co-stimulatory signals (e.g., CD80/CD86), while secreting cytokines such as IL-12, which collectively activate CD8⁺ T cells; the activated CD8⁺ T cells then recognize and attack cancer cells by releasing effector molecules like IFN-γ. (2) Immune Escape Mechanisms: Cancer cells evade T cell recognition by downregulating MHC-I expression, among other strategies, and recruit immunosuppressive cells such as M2-type macrophages and regulatory T cells (Tregs). These inhibitory immune cells contribute to an immunosuppressive microenvironment by secreting factors including CCL2, IL-6, and IL-10, thereby dampening the antitumor immune response and promoting tumor growth.

In tumor cells, OTUB1 indirectly modulates antitumor immune responses primarily by regulating cell death modalities and metabolic processes. OTUB1-mediated suppression of ferroptosis leads to significantly reduced release of damage-associated molecular patterns (DAMPs), such as HMGB1, ATP, and calreticulin [40]. The deficiency of these molecules impedes dendritic cell (DC) maturation, manifested as downregulation of costimulatory molecules (e.g., CD80/CD86), reduced IL-12 secretion, and ultimately impaired antigen presentation and CD8⁺ T cell activation [41]. Furthermore, by activating nodes such as p62, OTUB1 may enhance selective autophagy, potentially promoting the degradation of MHC class I molecules and associated antigen presentation machinery. However, this mechanism’s specific role in HCC remains to be directly supported, as current evidence largely derives from models of other cancers [42–44]. Concurrently, autophagy-activated tumor cells secrete factors such as CCL2 and IL-6, recruiting M2 macrophages and Tregs, thereby further shaping an immunosuppressive microenvironment [45, 46] (see Fig. 4).

### Direct functions of OTUB1 in immune cells

Within the HCC TIME, OTUB1 exerts distinct pathological functions through both cell-autonomous mechanisms in tumor cells and direct modulation of immune cell activity, collectively fostering an immunosuppressive landscape conducive to therapy resistance.

#### Direct modulation of T cell function in HCC

OTUB1 directly influences the development, activation, and function of various immune cells, critically shaping antitumor immune outcomes. In T cells, OTUB1 negatively regulates TCR–CD28 costimulatory signaling by deubiquitinating TRAF6 (cleaving K63-linked chains), thereby attenuating NF-κB activation and suppressing T cell activation [33, 47]. Additionally, OTUB1 stabilizes the PD-L1 protein and prolongs its half-life, enhancing inhibitory signaling toward T cells and accelerating T cell exhaustion programs [48].

#### Regulation of myeloid cell activity in HCC

OTUB1 significantly influences myeloid cell populations within the HCC microenvironment. While OTUB1’s role in HCC-associated macrophages requires further validation, studies in atherosclerosis demonstrate its ability to promote macrophage lipid accumulation and foam cell formation through SR-A stabilization [49], suggesting potential similar mechanisms in HCC TAMs. In dendritic cells, OTUB1 enhances NF-κB-driven pro-inflammatory cytokine production [37], which may paradoxically contribute to chronic inflammation that supports tumor progression.

#### Metabolic reprogramming of immune cells in HCC

A key aspect of OTUB1’s function in the HCC TIME involves immune-metabolic regulation. OTUB1 deubiquitinates NDUFS2, enhancing mitochondrial function and providing energetic support for immunosuppressive cells including Tregs and M2-polarized macrophages [50]. Additionally, OTUB1 stabilizes ACSL5, promoting acyl-CoA synthesis and conferring a lipid metabolic advantage that consolidates the immunosuppressive functions of these cells within the HCC microenvironment [51]. This highlights a crucial mechanism by which OTUB1 reshapes the TME: by directly reprogramming the metabolism of immune cells to favor their pro-tumorigenic functions. Investigating whether OTUB1 inhibition can reverse this metabolic reprogramming and restore anti-tumor immunity is a promising research avenue.

These OTUB1-mediated mechanisms collectively establish an immunosuppressive niche in HCC that not only facilitates immune evasion but also reinforces resistance to conventional and immunotherapeutic interventions. The multifaceted nature of OTUB1’s actions across different immune cell populations underscores its potential as a therapeutic target for reprogramming the HCC immune microenvironment.

## The central role of OTUB1 in driving HCC drug resistance: mechanisms and therapeutic targeting

### OTUB1 as a pivotal member of the OTU family with high translational potential

Within the OTU family, OTUB1 has emerged as a research focus due to its broad functional involvement and clear clinical relevance. It regulates ubiquitination through dual mechanisms: directly cleaving ubiquitin chains on substrates, and allosterically inhibiting ubiquitination by non-catalytically binding to E2 enzymes [52]. In multiple cancers (e.g., liver and breast cancer), upregulation of OTUB1 is significantly associated with poor patient prognosis, and its functions span proliferation, migration, immune evasion, and chemotherapy resistance [48, 53]. However, other OTU members also contribute to HCC drug resistance; OTUD1, for instance, promotes resistance by stabilizing TopBP1 in DNA damage repair, while OTULIN’s loss can exacerbate NF-κB-driven inflammation, fostering a resistant niche [25, 26]. Integrating targeting of multiple OTU members may thus yield synergistic effects.

### Stabilizing oncoproteins to support cell proliferation and apoptosis resistance

**Fig. 5:**
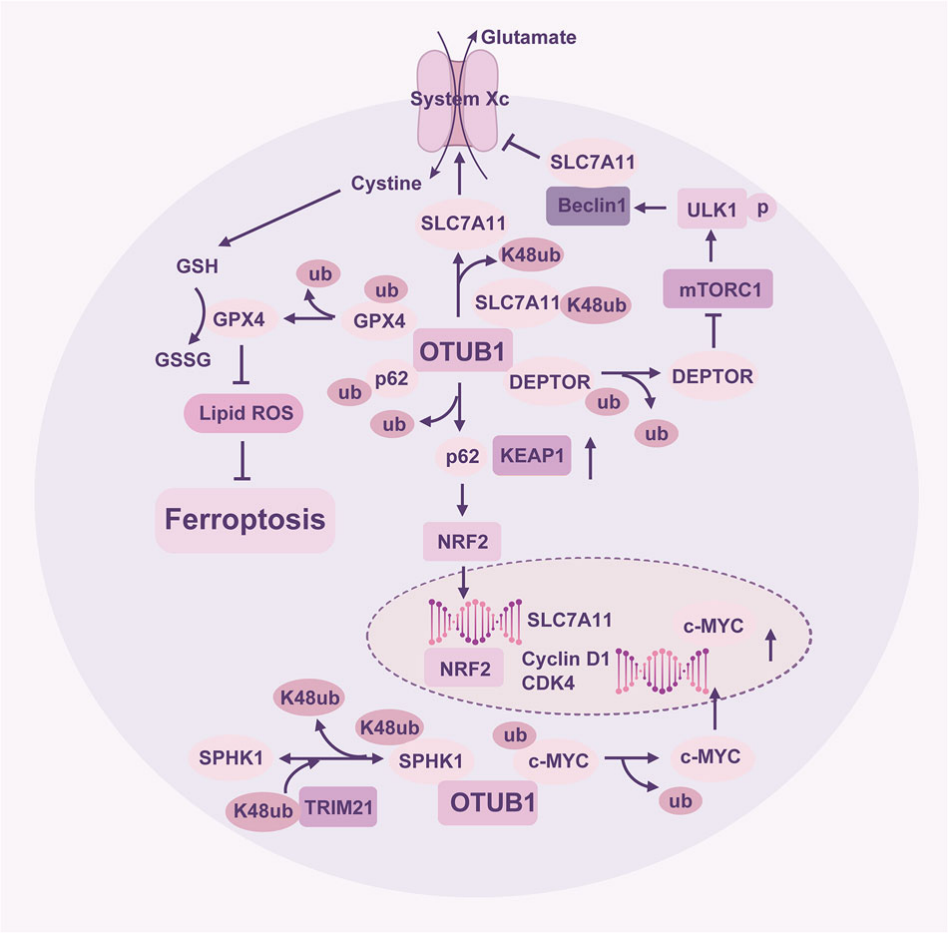
The core mechanisms by which OTUB1 drives drug resistance in HCC cells. This schematic integrates the key signaling pathways and molecular interaction networks that regulate ferroptosis. At the center of the figure is the process of ferroptosis itself, the sensitivity of which is precisely controlled by several core proteins. The main regulatory modules include: (1) The System Xc⁻-GPX4 axis: Cystine uptake mediated by SLC7A11 is crucial for glutathione (GSH) synthesis, and GPX4 utilizes GSH to reduce lipid peroxides, representing the primary defense line against ferroptosis; (2) The pivotal role of OTUB1: The deubiquitinating enzyme OTUB1 positively regulates this system by deubiquitinating and stabilizing both SLC7A11 and c-MYC, thereby suppressing ferroptosis; (3) The Keap1-NRF2 pathway: NRF2, acting as the master regulator of the antioxidant response, upregulates the expression of multiple cytoprotective genes, including SLC7A11, upon activation; (4) mTORC1 and autophagy: mTORC1 signaling, through associated molecules like its inhibitory protein DEPTOR and the selective autophagy adapter p62, indirectly influences cellular susceptibility to ferroptosis; (5) Transcriptional regulation by c-MYC: c-MYC can promote ferroptosis by repressing the transcription of SLC7A11. The arrows and connectors in the figure clearly illustrate the complex interactions between these molecules, including activation, inhibition, ubiquitination, and deubiquitination, ultimately revealing OTUB1 as a network hub that integrates signals from multiple pathways to determine cellular fate.

OTUB1 exerts its function primarily by stabilizing key oncoproteins, precisely regulating cell death pathways—particularly the crosstalk between ferroptosis and autophagy—and reprogramming cellular metabolism to counteract therapeutic stress, thereby collectively enabling HCC cells to escape conventional targeted therapies [54] (see Fig. 5).

OTUB1 directly interacts with multiple core oncoproteins and blocks their proteasomal degradation via deubiquitination, thereby enhancing tumor cell survival and proliferative capacity [55]. Studies have confirmed that OTUB1, through its deubiquitinase activity, directly removes ubiquitin chains from c-MYC, significantly prolonging its half-life and leading to abnormal nuclear accumulation of c-MYC. This results in sustained activation of downstream proliferation-related genes (such as Cyclin D1 and CDK4), driving cell cycle progression [56]. It has been shown that the E3 ubiquitin ligase TRIM21 promotes K48-linked polyubiquitination of SPHK1, accelerating its protein degradation. OTUB1 counteracts this process by deubiquitinating and stabilizing SPHK1, maintaining its high expression level in cells. Elevated SPHK1 expression, in turn, exacerbates malignant tumor progression through multiple signaling pathways [49].

### OTUB1 regulates the autophagy–ferroptosis axis and its mechanism in HCC drug resistance

#### The interplay between autophagy and ferroptosis in hepatocellular carcinoma

The crosstalk between autophagy and ferroptosis has emerged as a key research focus in understanding drug resistance mechanisms in HCC. These two cellular processes interact through shared molecular nodes, forming a complex regulatory network that profoundly influences the response of HCC cells to therapy [57]. A major open question is exactly how specific regulators like OTUB1 modulate autophagy in HCC and where this intersects with ferroptosis regulation.

Ferroptosis is an iron-dependent form of programmed cell death driven by the accumulation of lipid peroxides [58]. Its core molecular mechanism relies on the precise regulation of the System Xc⁻–GSH–GPX4 axis. SLC7A11, the light chain subunit of System Xc⁻, mediates cystine uptake—a rate-limiting step for glutathione (GSH) synthesis. GPX4 (glutathione peroxidase 4) utilizes GSH to reduce toxic lipid hydroperoxides (PL-OOH), thereby suppressing ferroptosis. The activity of this pathway directly determines cellular susceptibility to ferroptosis [59].

Autophagy plays a dynamic and dual role in HCC, with its function being highly context-dependent. On one hand, autophagy exerts pro-survival effects: by clearing damaged mitochondria and protein aggregates, it maintains intracellular metabolic homeostasis, thereby mediating resistance to targeted therapies such as sorafenib [60]. On the other hand, autophagy can also promote cell death: excessive activation of autophagy may lead to cellular self-digestion or facilitate ferroptosis through specific types of selective autophagy, such as ferritinophagy [61]. Ferritinophagy, mediated by receptors like NCOA4, releases labile iron and is a key amplifier of ferroptosis signaling, representing a critical point of intersection with OTUB1’s functions. p62/SQSTM1 and Beclin1 are core molecular nodes mediating these complex functions. As an autophagy receptor, p62/SQSTM1 not only targets ubiquitinated substrates to autophagosomes but also binds to Keap1, relieving its inhibition of NRF2. This promotes NRF2 nuclear translocation and subsequent transcription of antioxidant genes, including SLC7A11, providing a molecular basis for the interplay between autophagy and antioxidant defense [62]. Beclin1, a core component of the class III PI3K complex, is essential for autophagosome formation. Recent studies have also revealed that Beclin1 can bind to SLC7A11 and inhibit System Xc⁻ activity, uncovering a novel mechanism through which core autophagy machinery directly regulates ferroptosis execution [63]. This Beclin1-SLC7A11 axis is a prime candidate for exploration as a potential point of regulation by OTUB1 in HCC.

The regulation of ferroptosis by autophagy is precise and bidirectional. In promoting ferroptosis, autophagy enhances the intracellular labile iron pool through ferritinophagy (mediated by the receptor NCOA4), supplying the iron catalyst required for lipid peroxidation [64]. It also provides polyunsaturated fatty acids (PUFAs)—substrates for lipid peroxidation—via lipophagy-mediated breakdown of lipid droplets [65]. In suppressing ferroptosis, autophagy reduces the source of reactive oxygen species (ROS) by clearing dysfunctional mitochondria through mitophagy [66]. Moreover, sustained activation of the p62–NRF2 axis upregulates the expression of antioxidant genes such as SLC7A11, enhancing the overall cellular antioxidant capacity [67]. The OTUB1-p62-NRF2 axis may thus serve as a critical feedback loop fine-tuning the autophagy-ferroptosis balance, though its specific role in HCC requires direct validation.

#### Regulation of the autophagy–ferroptosis axis by OTUB1

OTUB1 exhibits high complexity and context-dependent functionality in regulating ferroptosis and autophagy. Studies demonstrate that OTUB1 directly binds to SLC7A11, a key component of System xc⁻, and removes its K48-linked ubiquitin chains, thereby preventing its proteasomal degradation. This action sustains cystine uptake and glutathione (GSH) synthesis, enhancing cellular antioxidant defense. In lenvatinib-treated HCC cells, knockdown of OTUB1 significantly shortens the half-life of SLC7A11 protein [68]. Similarly, OTUB1 has been shown to stabilize GPX4—the key enzyme that reduces toxic lipid peroxides and eliminates lethal lipid ROS—via deubiquitination [69]. Inhibition or knockdown of OTUB1 reduces protein levels of both SLC7A11 and GPX4, leading to significantly increased sensitivity to ferroptosis inducers such as erastin [68, 69]. This establishes OTUB1 as a central suppressor of ferroptosis, fostering chemoresistance.

However, current studies on OTUB1’s role in the autophagy-ferroptosis axis exhibit significant limitations. For instance, while OTUB1 suppresses ferroptosis by stabilizing SLC7A11 and GPX4, this mechanism is primarily validated in vitro and lacks confirmation in clinically heterogeneous HCC samples, particularly across etiologies like viral hepatitis or metabolic-associated fatty liver disease. More critically, the regulatory role of OTUB1 on p62/SQSTM1 remains unclear: although a study in myoblasts showed OTUB1 deubiquitinates and stabilizes p62 to activate the NRF2 pathway and indirectly suppress ferroptosis [44], this has not been directly verified in HCC, constituting a key unresolved node in the autophagy-ferroptosis-immune network. Future research must prioritize elucidating the OTUB1-p62 axis specificity in HCC—whether p62 stabilization not only affects ferroptosis susceptibility but also indirectly reshapes the immune microenvironment by modulating secretion of factors like CCL2 and IL-6. Additionally, OTUB1’s interaction with Beclin1 may indirectly influence autophagy via the mTORC1-DEPTOR pathway [70, 71], but whether this intersection drives immune evasion remains unclear, highlighting the network’s complexity. These limitations underscore that OTUB1-targeting strategies must account for its pleiotropic effects to avoid oversimplification.

Furthermore, OTUB1 modulates autophagic flux through key regulators such as p62/SQSTM1 and Beclin1, thereby engaging in crosstalk with ferroptosis. It should be noted that a study in myoblasts showed OTUB1 deubiquitinates and stabilizes p62, enhancing its interaction with KEAP1 and promoting NRF2 activation and subsequent transcription of antioxidant genes, which indirectly suppresses ferroptosis [44]. However, this mechanism has not been directly validated in liver cancer, representing an important direction for future research. Specifically, it is crucial to determine if the OTUB1-p62 axis in HCC cells not only impacts ferroptosis susceptibility but also influences the secretion of immunomodulatory factors like CCL2 and IL-6, thereby indirectly shaping the immune microenvironment. Additionally, OTUB1 can deubiquitinate and stabilize DEPTOR, leading to inhibition of mTORC1 activity. Subsequent release of autophagy inhibition activates the ULK1 complex, which phosphorylates and activates the Beclin1–class III PI3K complex to initiate autophagosome formation, thereby indirectly influencing ferroptosis [70, 71]. The potential for OTUB1 to regulate the Beclin1-SLC7A11 interaction directly remains an open and mechanistically significant question.

In summary, OTUB1 drives the development of drug resistance in HCC by stabilizing oncoproteins such as c-MYC and fine-tuning cell death modalities—such as suppressing ferroptosis via the SLC7A11/GPX4 axis. These mechanisms are interrelated and collectively delineate a complex network, positioning OTUB1 as a promising therapeutic target in HCC treatment. The development of specific inhibitors targeting OTUB1 may offer novel strategies to reverse drug resistance in HCC (see Fig. 5).

It is noteworthy that there is a significant scarcity of research on the correlation between the OTU deubiquitinase family and autophagy, particularly focusing on members other than OTUB1 (e.g., OTUD3, OTULIN, and OTUD6B). This limitation may stem from the early stage of investigation in this field: on one hand, the functional diversity and substrate specificity of OTU family members pose challenges for systematically exploring their interactive networks with autophagy pathways; on the other hand, existing literature predominantly concentrates on prominent molecules like OTUB1, resulting in the oversight of other members’ roles in autophagy regulation. Despite the paucity of studies, the rationale for discussing this area lies in its potential translational value—autophagy, as a core process in HCC drug resistance, is intimately linked to the deubiquitination functions of the OTU family. Future research should leverage high-throughput screening, gene editing technologies (e.g., CRISPR-Cas9), or multi-omics analyses to systematically assess the roles of OTU members at critical autophagy nodes (such as the ULK1 complex or LC3 lipidation), thereby addressing knowledge voids and unveiling novel targets for combination therapies.

### Role of other OTU family members in HCC drug resistance

Beyond OTUB1, several OTU deubiquitinases influence HCC progression through distinct mechanisms. OTUD3 functions as a tumor suppressor by deubiquitinating and destabilizing HIF-1α, thereby inhibiting hypoxia-induced resistance to therapies like sorafenib [26]. Conversely, OTUD6A promotes chemoresistance by stabilizing oncoproteins such as CDK1, enhancing cell cycle progression [25]. In immune modulation, OTUD5 regulates macrophage polarization via CARD9, potentially contributing to immunosuppression in HCC [30]. Notably, these members often interact with OTUB1-driven pathways: for example, OTULIN’s inhibition of NF-κB may counterbalance OTUB1’s pro-survival signals, while OTUD3’s anti-angiogenic effects could synergize with OTUB1 blockade to exacerbate ferroptosis. These findings highlight that OTU family members operate in a network, where targeting multiple nodes—such as combining OTUB1 inhibition with OTUD3 agonists or OTULIN activators—could overcome compensatory resistance mechanisms and provide a more comprehensive therapeutic approach.

## Targeting OTUB1: strategies to reverse drug resistance and reprogram the immune microenvironment

As a key member of the OTU deubiquitinase family, OTUB1 plays a central role in HCC drug resistance and immune microenvironment remodeling by regulating the stability of multiple critical proteins through its deubiquitination activity. Its functions span both tumor cell–autonomous mechanisms and immunoregulatory pathways, making it an ideal target for combination therapy strategies. A paramount question is whether OTUB1 blockade can synergize with ferroptosis inducers or ICIs for greater therapeutic efficacy by directly modulating the autophagy-ferroptosis axis, which is central to HCC progression.

### Development of OTUB1 small-molecule inhibitors and innovative delivery strategies

Currently, several small-molecule inhibitors of OTUB1 have demonstrated promising antitumor efficacy in preclinical studies. For example, ACS607 specifically binds to the catalytic active site (Cys91) of OTUB1, effectively inhibiting its deubiquitinase activity. This promotes the degradation of proteins such as SLC7A11, GPX4, and c-MYC via the ubiquitin–proteasome pathway, thereby increasing tumor cell susceptibility to ferroptosis [68, 72]. Critically, these inhibitors directly disrupt the autophagy-ferroptosis balance by destabilizing SLC7A11 (a key component of System Xc- that regulates ferroptosis) and p62 (a mediator of selective autophagy), thereby synchronously inducing lipid peroxidation and autophagic flux dysregulation. Similarly, emerging inhibitors targeting other OTU members, such as OTUD3 or OTULIN, are under investigation; for instance, compounds that enhance OTUD3’s tumor-suppressive functions could complement OTUB1 blockade by reducing HIF-1α stability [26], which indirectly influences hypoxia-induced autophagy and ferroptosis sensitivity. Nanoparticle-based delivery systems can be adapted for multi-target approaches, improving specificity by co-delivering agents that concurrently target autophagy (e.g., Beclin1 modulators) and ferroptosis (e.g., GPX4 inhibitors).

However, given the baseline expression of OTUB1 in multiple normal tissues, systemic administration may lead to off-target toxicity. To improve therapeutic specificity, pH-sensitive liposomes (pH-Lipo) and functionalized nanoparticles have been extensively investigated. These carriers leverage the acidic nature of the TME (pH ~6.5) to achieve targeted drug release. Further enhancement of tumor accumulation is accomplished through surface modifications, such as cRGD peptides for targeting integrin αvβ3 or anti-GPC3 antibodies for recognizing HCC-associated antigens [73–77]. Recently, dual-specificity nano-systems co-loaded with an OTUB1 inhibitor and an immune modulator (e.g., a PD-L1 blocking antibody) have demonstrated simultaneous targeting of both tumor cells and the immune microenvironment. Preliminary results show significant tumor suppression and extended survival in humanized HCC mouse models [46, 78]. Importantly, these nanoplatforms can be engineered to release drugs in response to autophagy- or ferroptosis-associated biomarkers (e.g., elevated LC3-II or lipid ROS), ensuring precise spatial-temporal control over the core processes highlighted in the title.

However, therapeutic strategies targeting OTUB1 require systematic comparison of advantages and disadvantages to guide clinical translation. Small-molecule inhibitors (e.g., ACS607) offer high specificity by directly inhibiting OTUB1 catalytic activity, inducing degradation of SLC7A11/GPX4 [68, 72], but their disadvantage lies in potential off-target toxicity due to OTUB1’s basal expression in normal tissues (e.g., cardiovascular and liver), such as disrupting fatty acid oxidation [51]. Nano-delivery systems (e.g., pH-sensitive liposomes) enhance tumor specificity by targeting GPC3 or integrin αvβ3 [73–77], with advantages in reducing systemic toxicity, but drawbacks include complex preparation and potential immunogenic reactions. Combination therapies (e.g., OTUB1 inhibitor + ferroptosis inducer + immune checkpoint inhibitor) synergistically amplify efficacy through multi-node coordination, but the authors note that their advantages and disadvantages depend on sequence optimization: prior OTUB1 inhibition can reduce PD-L1 and antioxidant defenses, creating a permissive environment for subsequent therapies; however, this “triple combination” may exacerbate immune-related adverse events and high costs. Key unresolved nodes include whether OTUB1 blockade triggers compensatory upregulation of other DUBs (e.g., USP7) and how to dynamically monitor responses via biomarkers (e.g., p62 nuclear translocation or sPD-L1). This comparative analysis emphasizes that OTUB1-targeting strategies should be personalized, integrating tumor molecular subtypes and immune microenvironment features.

### Combination therapy strategies: inducing ferroptosis and immune activation

While targeting OTUB1 alone can partially suppress tumor progression, its greatest therapeutic potential lies in synergistic combination with existing treatment modalities. As elaborated in Sections 3 and 4, OTUB1 inhibition simultaneously enhances ferroptosis sensitivity and reverses immunosuppression, providing a strong rationale for combination therapies that directly engage the autophagy-ferroptosis axis.

#### Combination with ferroptosis inducers

Studies have demonstrated that combining OTUB1 inhibitors with a GPX4 inhibitor (e.g., RSL3) or a System Xc⁻ inhibitor (e.g., Erastin) significantly induces ICD. Tumor cells undergoing ICD release damage-associated molecular patterns (DAMPs), such as high mobility group box 1 (HMGB1) and ATP, which promote dendritic cell (DC) maturation and antigen presentation, thereby activating cytotoxic T cell responses [12, 41]. This strategy is intrinsically linked to ferroptosis execution, as OTUB1 inhibition amplifies lipid peroxidation through SLC7A11/GPX4 destabilization, while concurrently modulating autophagy to prevent compensatory survival pathways. In immunocompetent HCC mouse models, this combination strategy not only suppressed primary tumor growth but also significantly reduced the number of distant metastases and induced an immune memory effect that protected against tumor recurrence [68, 69].

#### Combination with ICIs

Given that OTUB1 promotes T cell exhaustion by deubiquitinating and stabilizing PD-L1 [48], combining an OTUB1 inhibitor with an anti–PD-1/PD-L1 antibody is expected to produce synergistic effects: the OTUB1 inhibitor reduces tumor cell PD-L1 expression and alleviates T cell suppression, while immune checkpoint blockade (ICB) interrupts PD-1/PD-L1 interactions. Notably, this approach is reinforced by OTUB1’s role in the autophagy-ferroptosis network—for instance, OTUB1 inhibition can trigger autophagic degradation of PD-L1 and enhance ferroptosis-associated antigen release, creating a self-reinforcing cycle of immune activation. Together, they reverse T cell exhaustion through complementary mechanisms. Single-cell sequencing analyses further revealed that combination therapy enriches a population of stem-like precursor exhausted T cells (TPEX cells). This subset exhibits enhanced proliferative and effector potential and may be critical for long-term immune surveillance [78].

#### Innovative triple-combination therapeutic strategy: mechanistic synergy beyond additive effects

The triple-combination regimen—integrating an OTUB1 inhibitor, a ferroptosis inducer, and immune checkpoint blockade (ICB)—represents a sophisticated therapeutic approach designed to concurrently disrupt multiple vulnerability nodes in HCC. This strategy is meticulously aligned with the title’s focus on “coordinating the autophagy-ferroptosis balance,” as it sequentially targets: (1) OTUB1 to prime the autophagy-ferroptosis axis (e.g., by destabilizing SLC7A11 and p62), (2) ferroptosis inducers to amplify iron-dependent cell death, and (3) ICBs to harness immunogenic effects. Preclinical validation of this synergy is critical. Rather than merely combining three distinct mechanisms, this strategy establishes a mutually reinforcing cycle that progressively dismantles tumor defense systems and augments antitumor immunity through sequential physiological conditioning.

Priming the TME via OTUB1 Inhibition. OTUB1 serves as a critical resistance node by simultaneously stabilizing PD-L1 and key ferroptosis defense proteins (SLC7A11/GPX4). Its inhibition creates a permissive environment for subsequent therapies through two primary mechanisms: First, by destabilizing PD-L1, OTUB1 inhibition reduces immediate immune inhibitory pressure, thereby lowering the threshold for T-cell reactivation upon ICB administration [48]. Second, through degradation of SLC7A11 and GPX4, OTUB1 inhibition depletes the antioxidant defense system, sensitizing tumor cells to ferroptosis inducers that would otherwise require higher concentrations to overcome basal resistance [68, 69]. This preconditioning effect is further amplified by OTUB1’s regulation of autophagy, which can shift cellular fate toward ferroptosis vulnerability by disabling adaptive autophagy.

Amplification Through Sequential Stress Induction. The strategic administration sequence creates a feedforward loop of ICD. As ferroptosis inducers activate following OTUB1 inhibition, they trigger extensive lipid peroxidation in now-vulnerable tumor cells. This not only directly eliminates tumor cells but also generates massive damage-associated molecular patterns (DAMPs) [12, 40]. The released ATP and HMGB1 serve as potent dendritic cell (DC) activation signals, while exposure of calreticulin enhances phagocytic clearance. Consequently, DCs undergo maturation with upregulated antigen presentation capacity, priming naive T cells against tumor antigens released during ferroptosis. This process is intrinsically tied to autophagy modulation, as OTUB1 inhibition may concurrently disrupt autophagic flux, preventing the clearance of damaged mitochondria and amplifying ROS-driven ferroptosis.

Immune Activation and Sustained Pressure. ICB administration capitalizes on this primed immune environment. With reduced PD-L1-mediated suppression and increased tumor antigen availability, anti-PD-1/PD-L1 antibodies effectively unleash pre-activated T cells, converting them into potent cytotoxic effectors [78]. The simultaneous ferroptosis induction creates continuous antigen release, maintaining immune activation while preventing the outgrowth of resistant clones. Particularly significant is the observed enrichment of stem-like precursor exhausted T cells (TPEX cells), which possess enhanced proliferative capacity and durability—critical for long-term immune surveillance and preventing relapse.

Overcoming Compensatory Resistance Mechanisms. The triple combination strategically addresses typical escape pathways. While OTUB1 inhibition alone might trigger upregulation of alternative DUBs (e.g., USP7) or activation of NRF2 signaling [68, 70], the concurrent application of ferroptosis inducers and ICB creates simultaneous pressure across multiple systems. Tumor cells attempting to compensate through one pathway remain vulnerable to attack via the others, effectively closing escape routes that would emerge under monotherapy or dual-combination approaches.

This multi-mechanistic synergy demonstrates profound and durable treatment responses across various drug-resistant HCC models, including patient-derived xenografts with inherent heterogeneity. The approach is particularly suited for advanced HCC characterized by high GPC3 expression or intrahepatic metastasis—contexts where sequential therapeutic failure typically occurs due to rapid adaptation [54, 73].

### Immune cell-specific interventions and novel directions in cell therapy

The role of OTUB1 in immune cells suggests its potential as a target for optimizing cell-based therapies. In CAR-T cell therapy, CRISPR/Cas9-mediated knockout of OTUB1 enhances T cell activation and cytokine secretion (e.g., IFN-γ and TNF-α), while also improving in vivo persistence and tumor-infiltrating capacity [50]. Similarly, in adoptive macrophage therapy, targeted delivery of OTUB1 siRNA to TAMs using mannosylated nanoparticles promotes their repolarization toward the M1 phenotype, enhancing phagocytic function and antitumor immunity [30, 49].

### Broader therapeutic strategies involving the OTU family

To address the limitations of single-target therapies, future strategies should encompass the entire OTU family. For example, dual inhibitors targeting both OTUB1 and OTUD6A could simultaneously disrupt proliferation and immune evasion, while nano-delivery systems co-loaded with OTULIN agonists may reverse NF-κB-mediated resistance [25, 26]. Preclinical models show that combining OTUB1 inhibition with OTUD3 activation reduces tumor growth more effectively than monotherapy, highlighting the synergy within the OTU network. Critically, these multi-target approaches should be designed to explicitly coordinate the autophagy-ferroptosis balance—e.g., by simultaneously inhibiting OTUB1 (to suppress GPX4 and induce ferroptosis) and activating OTUD3 (to destabilize HIF-1α and modulate hypoxia-induced autophagy). Additionally, OTUD6B’s role in T cell recruitment suggests that its modulation could enhance checkpoint inhibitor efficacy. This integrated approach aligns with the title’s emphasis on “Targeting the OTU Family,” advocating for a shift from OTUB1-centric strategies to multi-member targeting for robust and durable responses in HCC, with a core focus on rescuing the dysregulated autophagy-ferroptosis axis.

## Discussion

### Current challenges: off-target toxicity and tissue-specific vulnerabilities

The core limitation of current OTUB1 research lies in its broad physiological functions, posing challenges for targeted therapy in terms of safety and specificity. For example, OTUB1 regulates metabolic homeostasis in normal hepatocytes [51], and its inhibition may disrupt lipid metabolism, while its dual roles in immune cells (e.g., T cells and macrophages) [33, 37] make it difficult to balance antitumor immunity with autoimmune risks through single-target approaches. The authors contend that future studies should focus on unresolved network nodes: how OTUB1 precisely modulates the autophagy-ferroptosis intersection (e.g., via Beclin1-SLC7A11 interactions) and its spatiotemporal dynamics in TME metabolic reprogramming. Furthermore, synergistic targeting of other OTU family members (e.g., OTUD3 and OTULIN) may overcome the limitations of OTUB1 monotherapy, but comparative analysis shows efficacy highly depends on tumor heterogeneity. In summary, OTUB1-targeting strategies must integrate multi-omics data and advanced models (e.g., organoids) to address limitations and optimize combination therapies.

The translational potential of OTUB1-targeted therapies is constrained by significant safety concerns stemming from OTUB1’s integral roles in physiological processes across multiple organ systems. A major hurdle is the development of highly selective OTUB1 inhibitors to minimize on-target toxicity in normal tissues. Beyond its established functions in tumor progression, OTUB1 maintains cellular homeostasis in several critical tissues where inhibition could precipitate adverse effects. In cardiovascular tissue, OTUB1 regulates the TRAF6-ASK1 signaling axis, with demonstrated protective functions against inflammatory stress in non-alcoholic steatohepatitis models [33]. Hepatic OTUB1 participates in fatty acid oxidation through ACSL5 stabilization, suggesting potential metabolic disturbances upon systemic inhibition [51]. Neurological functions may also be compromised given OTUB1’s regulation of mitochondrial proteins like NDUFS2 in pancreatic cells, a mechanism likely conserved in neuronal populations [50]. These tissue-specific vulnerabilities underscore the necessity of advanced delivery systems capable of spatial control.

Nanoparticle-based strategies offer promising solutions to these challenges. pH-sensitive liposomes functionalized with HCC-specific ligands (e.g., anti-GPC3 antibodies) demonstrate preferential accumulation in tumor tissues through enhanced permeability and retention effects combined with active targeting [62, 74, 75]. Surface modifications with cyclic RGD peptides further improve tumor selectivity by exploiting integrin αvβ3 overexpression in HCC neo-vasculature [76]. Recent advances in dual-ligand systems achieve even greater specificity, as demonstrated by folate-integrin targeted nanoparticles showing 5.3-fold higher tumor accumulation compared to non-targeted counterparts in metastatic models [78]. These approaches collectively mitigate off-target risks while maintaining therapeutic efficacy at tumor sites.

### Advanced modeling systems for therapeutic evaluation

The complexity of OTUB1’s dual roles in tumor cells and immune compartments necessitates sophisticated preclinical models that accurately recapitulate human pathophysiology. Liver-on-a-chip platforms incorporating patient-derived hepatocytes, hepatic stellate cells, and endothelial cells enable real-time monitoring of OTUB1 inhibition effects on metabolic coupling and cytokine secretion [72]. These microfluidic systems can be further enhanced by integrating immune components, allowing evaluation of checkpoint inhibitor interactions within biomechanically relevant microenvironments.

Patient-derived organoids (PDOs) co-cultured with autologous immune cells represent another transformative approach. HCC PDOs maintain parental tumor genetic profiles and drug response patterns, while the addition of tumor-infiltrating lymphocytes enables assessment of OTUB1-targeted therapy on immune cell trafficking and cytotoxicity [48, 69]. Such models have already demonstrated predictive value for immunotherapy responses, with PDOs exhibiting correlated sensitivity to anti-PD-1 treatment matching patient clinical outcomes. Incorporating macrophage populations further allows investigation of polarization shifts following OTUB1 inhibition, addressing its dual impact on innate and adaptive immunity [30, 36].

### Clinically actionable biomarkers for patient stratification

Translating OTUB1-directed therapies into clinical practice requires robust biomarkers that reliably predict treatment response. The development of such predictive biomarkers is essential for enabling precision treatment in HCC. Immunohistochemical detection of OTUB1 protein levels in tumor biopsies provides a straightforward stratification metric, with studies showing ≥2.5-fold overexpression correlating with reduced overall survival in HCC cohorts [48, 53]. This simple assay can be implemented in most pathology laboratories, facilitating rapid patient selection.

Nuclear accumulation of p62/SQSTM1 serves as a functional biomarker indicating OTUB1 activity, as OTUB1-mediated stabilization promotes p62 translocation and NRF2 pathway activation [44, 62]. This readily detectable histological feature identifies tumors dependent on OTUB1-mediated antioxidant responses, potentially predicting sensitivity to ferroptosis-inducing combinations. Plasma-based biomarkers offer non-invasive monitoring alternatives, with soluble PD-L1 (sPD-L1) levels showing promise as dynamic indicators of target engagement. Elevated baseline sPD-L1 concentrations (>1.5 ng/mL) correlate with poor ICI response, while rapid reduction following OTUB1 inhibition may indicate successful pathway modulation [48, 78].

Emerging multi-analyte signatures incorporating ferroptosis-related genes (FSP1, SLC7A11) with immune markers (CD8^+^ T cell density) provide enhanced predictive power. Such composite scores account for tumor-intrinsic resistance mechanisms and microenvironmental context, enabling personalized therapy selection based on comprehensive molecular profiling [69, 73].

## Conclusion

This review systematically elucidates the central role of the OTU deubiquitinase family in driving drug resistance and immunosuppression in HCC. While OTUB1 stabilizes key proteins such as c-Myc, SLC7A11, and GPX4 to suppress ferroptosis and promote immune escape, other members like OTUD3, OTULIN, and OTUD6B contribute critically to HCC progression through distinct pathways. The synergistic or antagonistic interactions between OTU members—such as OTUD3’s anti-angiogenic effects and OTULIN’s anti-inflammatory properties—reveal a complex regulatory network that extends beyond OTUB1. To resolve the perceive disconnect between strategies and core processes, therapeutic designs must prioritize direct engagement with the autophagy-ferroptosis axis—for instance, by developing biomarkers that dynamically monitor autophagic flux (e.g., LC3 puncta) and ferroptosis activity (e.g., lipid peroxidation levels) in response to OTU-targeted therapies. Targeting the OTU family as a whole, through multi-inhibitor strategies or nano-delivery systems, demonstrates significant therapeutic potential for reshaping the TME and reversing resistance only if these strategies are rigorously anchored to the coordination of autophagy and ferroptosis. Future research must prioritize elucidating the crosstalk between OTU members in autophagy-ferroptosis balance and immune metabolism, ensuring that therapeutic designs align with the comprehensive scope implied by the title.
